# Supplementary figures and images for: Detecting the impact of land cover change on observed rainfall
Source: PeerJ. 2019 Aug 26;7:e7523. doi: 10.7717/peerj.7523 (PMC6715068; doi:10.7717/peerj.7523)

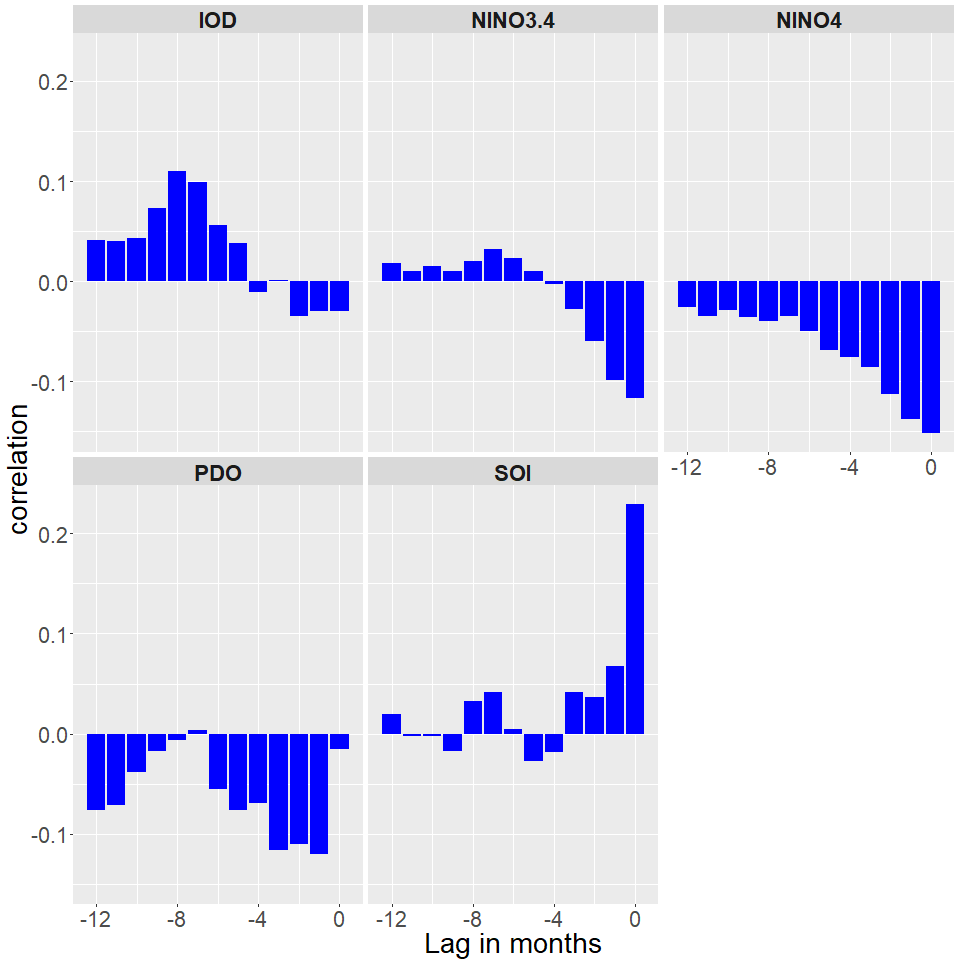

Supplement: Figure S1 — Bars in the plot indicate the strength of the cross-correlation at different lags. For the PDO analysis, 108-year rainfall data (1900–2008) are used. Otherwise, 36-year rainfall data are used. The correlation with NINO 3 is not shown as it is very similar to but weaker than for NINO 3.4. [file peerj-07-7523-s001.png]

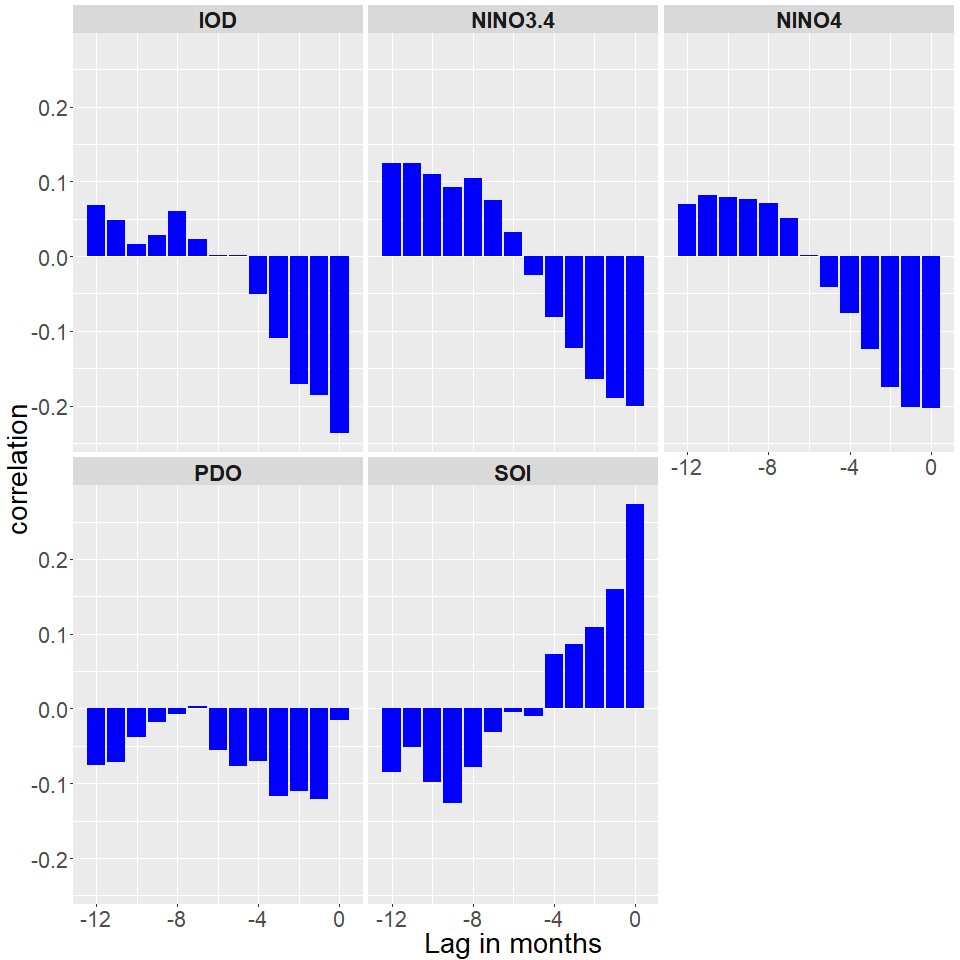

Supplement: Figure S2 — Bars in the plot indicate the strength of the cross-correlation at different lags. For the PDO analysis, 108-year rainfall data (1900–2008) are used. Otherwise, 36-year rainfall data are used. The correlation with NINO 3 is not shown as it is very similar to but weaker than for NINO 3.4. [file peerj-07-7523-s002.png]

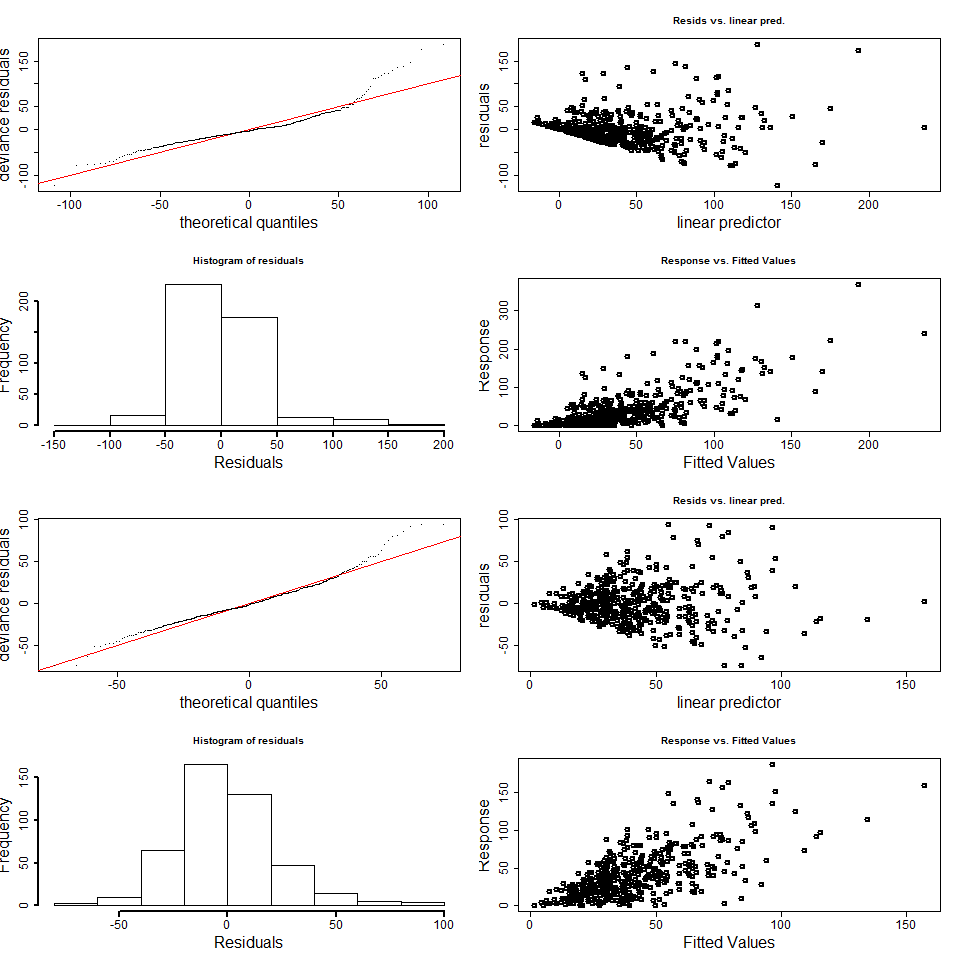

Supplement: Figure S3 — Results are shown for a sample pixel in the QLD region (top) and NSW/VIC region (bottom). Residuals are fairly normal as seen from the histogram and the qqplot, but show some scattering in the variance. [file peerj-07-7523-s003.png]

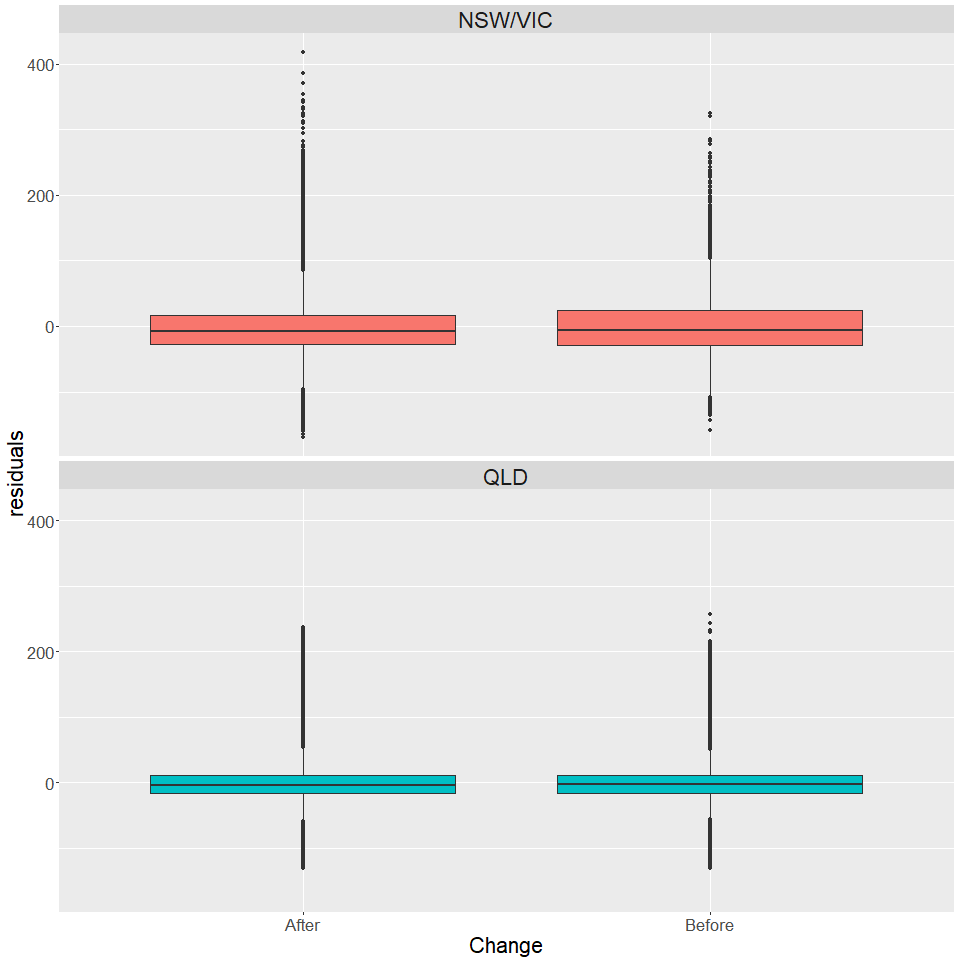

Supplement: Figure S4 — On average, the after period has a significantly lower annual rainfall residual in NSW/VIC, but a significantly higher annual rainfall residual in the Qld study area. [file peerj-07-7523-s004.png]
