# Supplementary material for: Detecting the impact of land cover change on observed rainfall
Source: PeerJ. 2019 Aug 26;7:e7523. doi: 10.7717/peerj.7523 (PMC6715068; doi:10.7717/peerj.7523)
Supplement: Supplemental Information 1 [file peerj-07-7523-s005.pdf]

Detecting the impact of land cover change on observed rainfall.

Chun Xia Liang[1], **Floris F. van Ogtrop**[1], **and R. Willem Vervoort**[1]

[1]**Sydney Institute of Agriculture, The University of Sydney, NSW 2006**

Corresponding author:
R. Willem Vervoort[1]

Email address: willem.vervoort@sydney.edu.au

ABSTRACT

This is the supplementary data for the paper "Detecting the impact of land cover change on observed rainfall." Peerj article number 35847

1 CODE FROM THE PAPER ON GITHUB

The Rmarkdown documents contain the code used in the analysis. These documents including most of the data and additional scripts are accessible via Github: https://github.com/WillemVervoort/RainfallLandcover/releases.
Please cite our paper if you plan to use any of the code.

2 SUMMARY OF DATA

This is a summary table of all the data used in the paper.

Table S 1. Summary of data.

**Data**	**Source**	**Resolution**		**Analysis period**
		Temporal	Spatial	
Percent tree cover	MOD44B	Annual	250m	2000-2010
Trend of vegetation cover change	DLCD (2009)	Onetime	250m	Trend of Apr 2000 - Apr 2008
Rainfall	AWAP gridded rainfall data	Monthly	$0.05° \times 0.05°$	Jan 1979- Dec 2008
SOI	BoM	Monthly	N/A	Jan 1979- Dec 2008
NINO 3, 3.4, 4	IRI/LDEO data library	Monthly	N/A	Jan 1979- Dec 2008
PDO	NOAA	Monthly	N/A	Jan 1979- Dec 2008
IOD	POAMA-2 dataset	Monthly	N/A	Jan 1979- Dec 2008

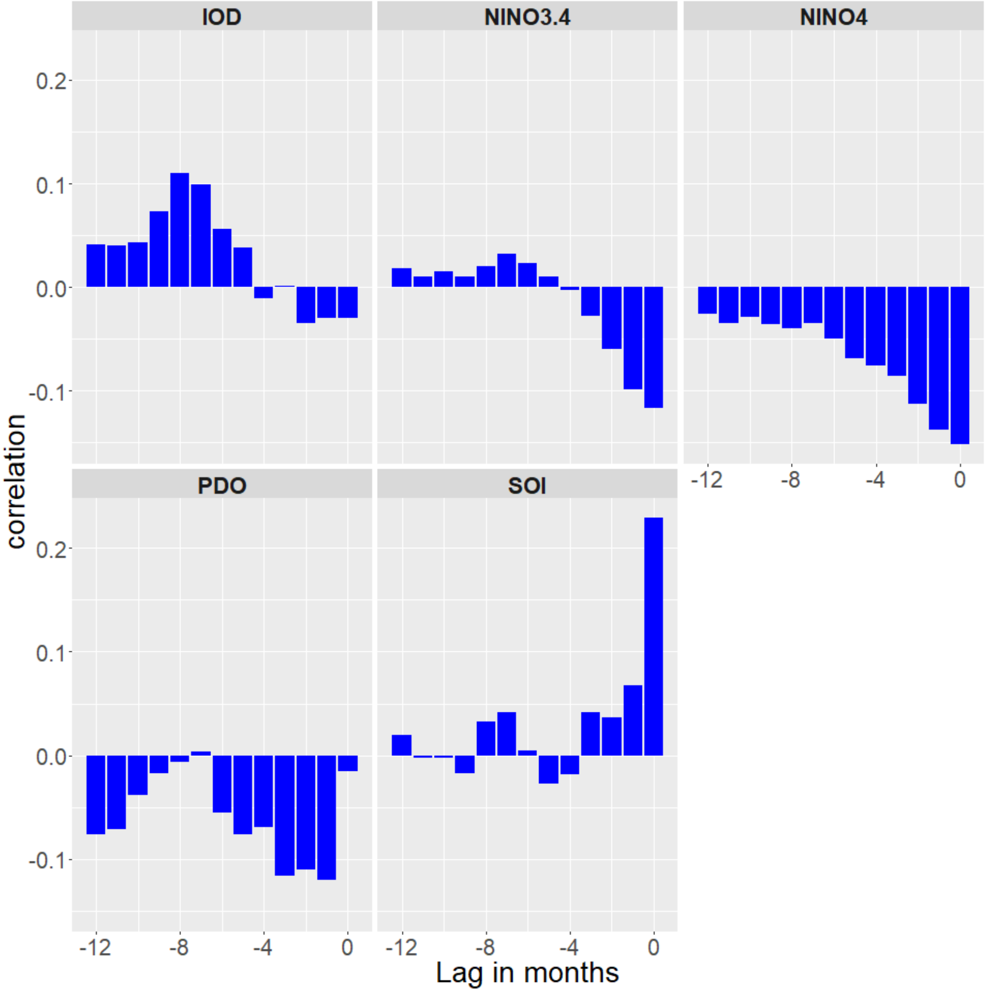

Figure S 1. Cross-correlation of six climate indices and rainfall in QLD study region. Bars in the plot indicate the strength of the cross-correlation at different lags. For the PDO analysis, 108-year rainfall data (1900 - 2008) are used. Otherwise, 36-year rainfall data are used. The correlation with NINO 3 is not shown as it is very similar to but weaker than for NINO 3.4.

3 CROSS CORRELATIONS RAINFALL AND CLIMATE INDICES

These figures were moved to the supplementary data during the review process.

Based on the correlation between the climatic indices and rainfall in the regions (as shown in Figure S 1 and Figure S 2), it can be concluded that:

- In QLD, the correlation between rainfall and SOI at zero time lags is the strongest across all indices, outweighing the other ENSO indicators. IOD and PDO have a weak influence in QLD.

- In NSW/VIC, again the SOI has the strongest correlation with rainfall, followed by the IOD. For both, the strongest correlations occur at the zero time lags.

- In both cases PDO had the weakest correlations, and this factor was therefore dropped as a predictor.

- In general, for the better correlated indices, strongest correlations occured at zero time lags.

Although some indices are serially correlated with rainfall up to several months, the lag zero events have the greatest correlation coefficients. Furthermore, using multiple climatic index series was generally found most useful in rainfall prediction (e.g. Risbey et al. 2009; Kamruzzaman, Beecham, and Metcalfe

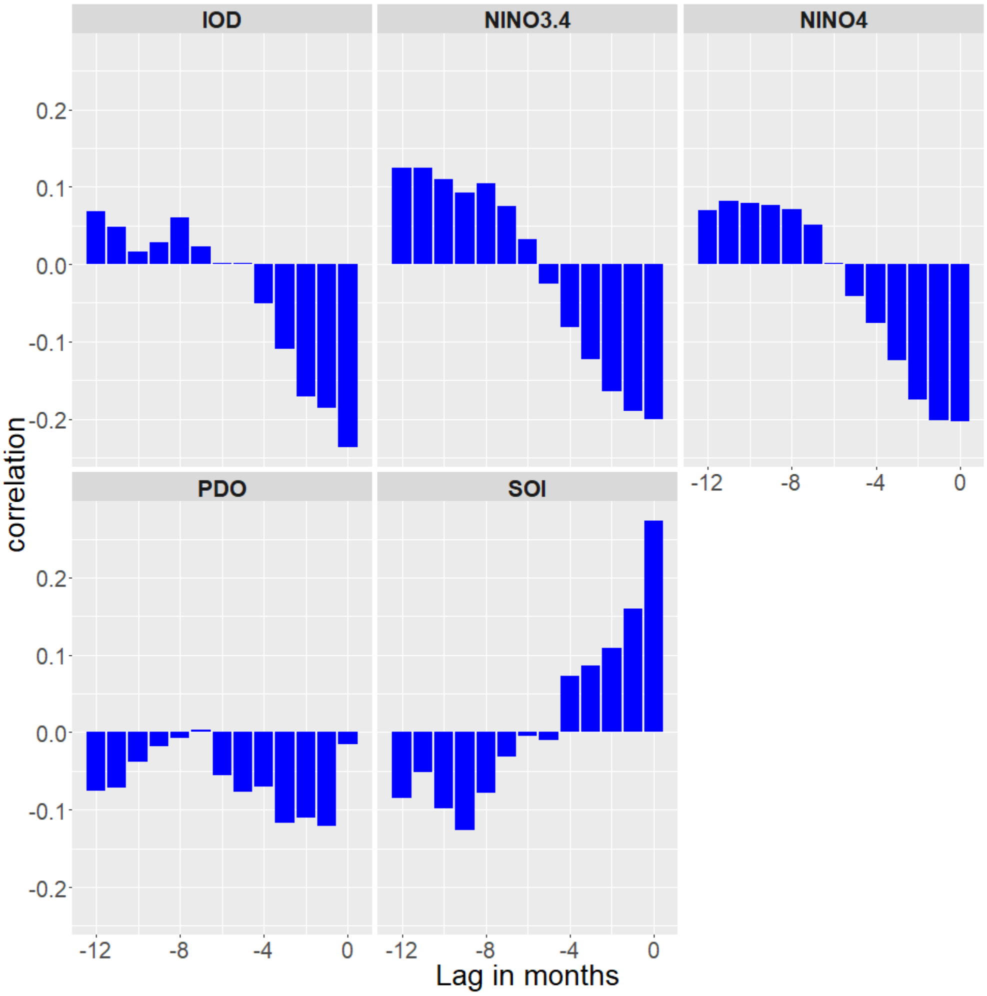

Figure S 2. Cross-correlation of six climate indices and rainfall in NSW/VIC study region. Bars in the plot indicate the strength of the cross-correlation at different lags. For the PDO analysis, 108-year rainfall data (1900 - 2008) are used. Otherwise, 36-year rainfall data are used. The correlation with NINO 3 is not shown as it is very similar to but weaker than for NINO 3.4.

2011).

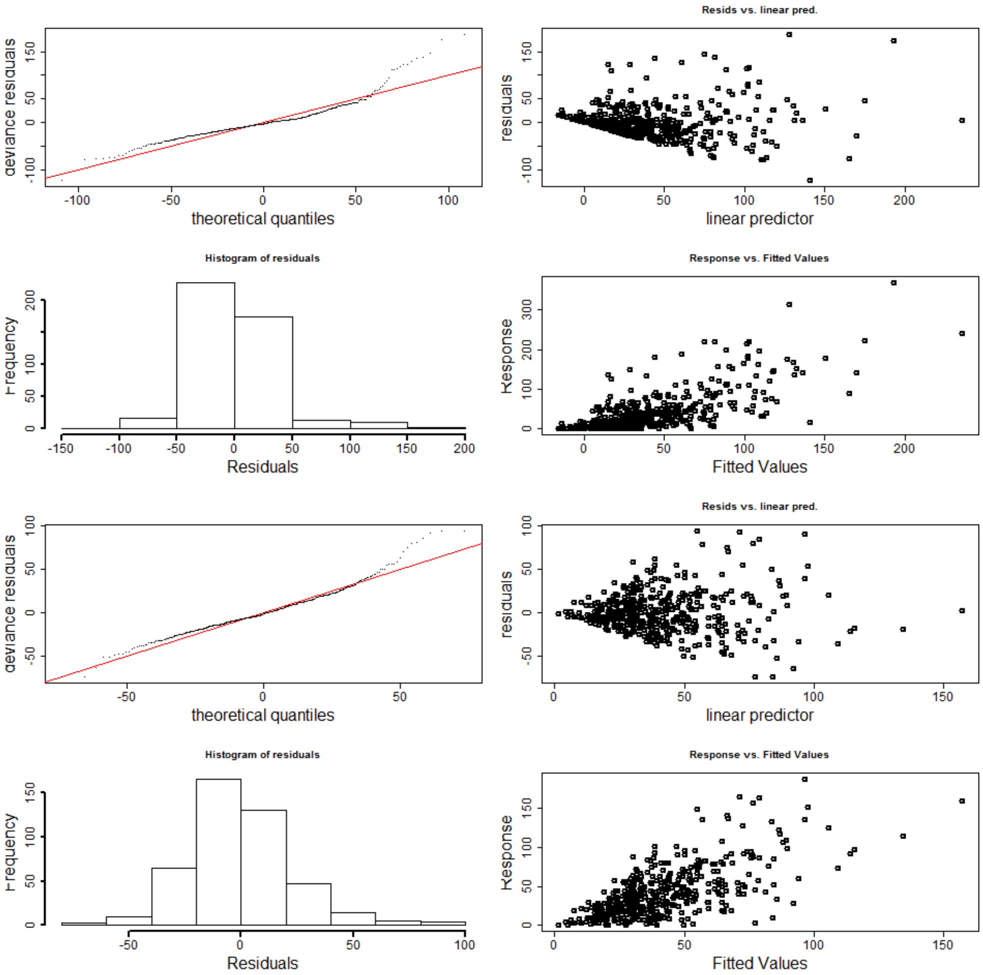

Figure S 3. The residual analysis of the Generalised additive modelling indicating how well the residuals follow the regression assumptions. Results are shown for a sample pixel in the QLD region (top) and NSW/VIC region (bottom). Residuals are fairly normal as seen from the histogram and the qqplot, but show some scattering in the variance.

4 FURTHER FIGURES

These two plots (Figure S 3) highlight the GAM residual analysis for a sample pixel. Residuals for other pixels were similar across the regions analysed.

Finally, Figure S 4 indicates a boxplot of the annual rainfall residuals across the regions.

REFERENCES

Kamruzzaman, M., S. Beecham, and A. V. Metcalfe. 2011. "Non-Stationarity in Rainfall and Temperature in the Murray Darling Basin." *Hydrological Processes* 25 (10). John Wiley & Sons, Ltd.: 1659–75. `http://dx.doi.org/10.1002/hyp.7928`.

Risbey, James S., Michael J. Pook, Peter C. McIntosh, Matthew C. Wheeler, and Harry H. Hendon. 2009. "On the Remote Drivers of Rainfall Variability in Australia." *Monthly Weather Review* 137 (10): 3233–53. `http://dx.doi.org/10.1175/2009MWR2861.1`.

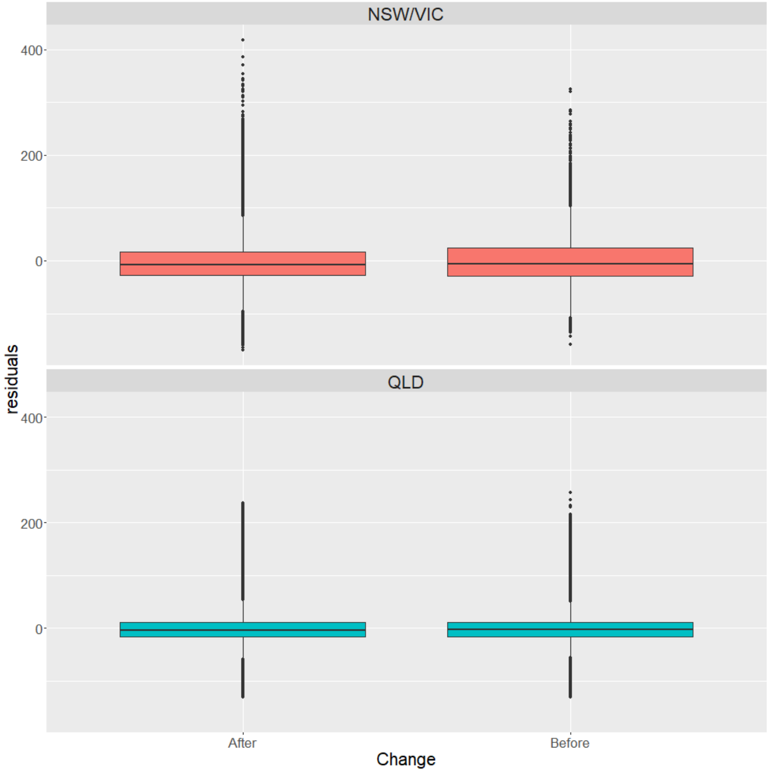

Figure S 4. Boxplots of annual rainfall residuals (estimated based on Equation 2 before and after the land cover intervention during 1979 - 2015 in the study regions. On average, the after period has a significantly lower annual rainfall residual in NSW/VIC, but a significantly higher annual rainfall residual in the Qld study area
